# Segregating BC_2_F_1_ interspecific hybrids between *Brassica napus* and *B. nigra* reveal a major effect locus for blackleg resistance on chromosome B2

**DOI:** 10.1007/s11032-026-01690-5

**Published:** 2026-07-04

**Authors:** Paula Vasquez-Teuber, Roman Gaebelein, Jessica L. Soyer, Thierry Rouxel, Jacqueline Batley, Annaliese S. Mason

**Affiliations:** 1 Department of Plant Breeding, Justus Liebig University, Heinrich-Buff-Ring 26-32, Giessen, 35392 Germany; 2 Department of Plant Production, Faculty of Agronomy, University of Concepción, Av. Vicente Méndez 595, Chillán, Chile; 3 Plant Breeding Department, University of Bonn, INRES, Kirschallee 1, Bonn, 53115 Germany; 4 Université Paris-Saclay, INRAE, UR BIOGER, Palaiseau, 91120 France; 5 Institut für Züchtungsforschung an landwirtschaftlichen Kulturen, Julius-Kühn- Institut, Rudolf-Schick-Platz 3a, OT Groß Lüsewitz, Sanitz, 18190 Germany; 6 School of Biological Sciences, University of Western Australia, Perth, WA 6009 Australia

**Keywords:** *Leptosphaeria maculans*, Disease resistance, Introgression breeding, *Brassica*, Interspecific hybridisation

## Abstract

Blackleg (Phoma, *Leptosphaeria maculans*) is one of the most important diseases affecting rapeseed (*Brassica napus*, 2n = AACC) worldwide. Although a number of major blackleg resistance genes have been identified in *B. napus*, finding novel sources of resistance is critical in dealing with this rapidly evolving fungal pathogen. Black mustard (*Brassica nigra*, 2n = BB) is a highly resistant wild relative species for which at least one blackleg resistance gene has been described (*Rlm10* on chromosome B4), but which has been rarely investigated as a source of blackleg resistance for introgression breeding (probably because it is self-incompatible, heterozygous, difficult to hybridize with *B. napus* and until recently lacked the genetic and genomic resources available for the *Brassica* A- and C-genome species). We produced allohexaploid hybrids (2n = AABBCC) between *B. napus* and *B. nigra* followed by two generations of backcrossing to develop a BC_2_F_1_ population segregating for inheritance of specific B-genome chromosomes from *B. nigra*. This population was phenotyped for blackleg resistance using a cotyledon test with blackleg isolate “JN2” (*AvrLm4-7*,* 5–9*,* 6*,* 8*,* 10 A-B*,* 11*,* S-Lep2*) and genotyped with B-genome chromosome-specific markers. Resistance segregated with the presence of chromosome B2 (*p* < 0.0001). Future work will target the identification or production of introgression lines, and recovery of this genetic locus from the B genome in a rapeseed (2n = AACC) genomic background. Our work highlights the potential of *B. nigra* and *Brassica* wild relatives as sources of novel biotic stress resistances.

**Supplementary Information:**

The online version contains supplementary material available at 10.1007/s11032-026-01690-5.

Rapeseed (*Brassica napus*) is the third most important oil crop worldwide (16% of oil production), after soybean and palm oil, and average yield has increased from 1.7 tonnes per hectare in 1981 to 2.6 tonnes per hectare in 2022 (FAO 2023). Major rapeseed producers include Canada, Australia, China, India, Germany, and France (FAO 2023). Rapeseed yields are negatively affected by blackleg (also known as Phoma) disease, which occurs worldwide and affects a range of *Brassica* crops (Rouxel and Balesdent 2005). This disease is caused by two *Leptosphaeria* species, *L. maculans* and *L. biglobosa*. These fungal species can be found around the world, and in countries where this crop is produced, such as Australia, Canada and Western Europe (Zhang and Fernando 2018), *L. maculans* is the most commonly recorded species (West et al. 2001). Yield losses in Australia due to *L. maculan*s are estimated at about 10 to 15% per year, and in some locations can be higher than 90% (Wouw et al. 2016).

Breeding rapeseed for resistance is one of the best strategies to control blackleg disease. To date, at least 19 major resistance genes have been identified within *Brassica* species, and five genes have been cloned: *LepR3*, *Rlm2*, *Rlm4*, *Rlm7*, and *Rlm9* (Yu et al. 2005, Yu et al. 2008, Yu et al. 2013, Delourme et al. 2006, Wouw et al. 2009, Long et al. 2011, Larkan et al. 2013, Larkan et al. 2015, Larkan et al. 2020, Raman et al. 2016, Raman et al. 2021, Degrave et al. 2021, Haddadi et al. 2022, Jiquel et al. 2021). Most resistance genes described to date are located in the A genome of *B. napus* or its progenitor species *B. rapa*, such as *Rlm1*, *Rlm2* (Ansan-Melayah et al. 1998), *Rlm3* (Delourme et al. 2004), *Rlm8* (Balesdent et al. 2002), *Rlm11* (Balesdent et al. 2013), *LepR1* (Yu et al. 2005), *RlmS/LepR2* (Yu et al. 2005, Wouw et al. 2009), and the allelic versions *Rlm4-Rlm7-Rlm9* (Larkan et al. 2020, Haddadi et al. 2022, Delourme et al. 2004, Balesdent et al. 2002), and *RLm2-LepR3* (Larkan et al. 2015, Ansan-Melayah et al. 1998, Li and Cowling 2003). Fewer resistance genes have been found in the C genome of *B. napus* and its progenitor species *B. oleracea*, with only one mapped gene (*Rlm13*) to date on chromosome C3 in *B. napus*, which may also be of A genome origin (Raman et al. 2021). Resistance has also been found in Korean genotypes of *B. oleracea* var. *capitata* (Robin et al. 2017), as well as a putative *Rlm14* gene in a Mexican cultivar (Degrave et al. 2021). In addition, 12 putative *Rlm* genes were found in *B. oleracea* within a syntenic region of *LepR4* (Ferdous et al. 2020). Resistance has also been observed in the wild relative *Brassica insularis*, a coenospecies of *B. oleracea* (Mithen and Lewis 1988), and in the C genome of the allotetraploid species *Brassica carinata* (Rahman et al. 2007). However, resistance gene breakdown is common, and the majority of resistance genes described to date are not effective against all isolates of the pathogen (Rouxel et al. 2024). For example, *Rlm1* was overcome within a five-year period in France (Rouxel et al. 2003) and *LepR3*, which is a functional equivalent of *Rlm1*, within three years in Australia (Sprague et al. 2006). Other examples of resistance breakdown are *Rlm1* in Australia (Van De Wouw et al. 2014), *Rlm2* in France (Rouxel and Balesdent 2017), *Rlm3* in Canada (Zhang et al. 2016), *Rlm6* in an experimental setting in France (Brun et al. 2010), *Rlm7* in the UK and France (Mitrousia et al. 2018, Balesdent et al. 2022) and *LepR1* in Australia (Sprague et al. 2006). Thus, identification of novel sources of resistance genes is of critical importance for ongoing breeding efforts to produce resistant rapeseed germplasm.

So far, three resistance genes (*Rlm5*, *Rlm6* and *Rlm10*) have been reported in the *Brassica* B genome based on typical gene-for-gene interactions with fungal isolates carrying the matching avirulence genes *AvrLm5*, *AvrLm6 AvrLm10* (Vasquez-Teuber et al. 2024). The *Brassica* B genome is not present in *B. napus* but is present in related crop species *Brassica nigra* (2n = BB), *B. juncea* (2n = AABB) and *B. carinata* (2n = BBCC). *Rlm5* (chromosome location unknown) (Balesdent et al. 2002) and *Rlm6* (or *Jlm1*) (Chèvre et al. 1997) both originate in *B. juncea* (2n = AABB), and *Rlm6* has been mapped to the *B. juncea* reference genome (Yang et al. 2016) on chromosomes A07 and B04 (Yang et al. 2021), although in silico integration of markers from old linkage maps suggested *Rlm6* was located on chromosome B01 (Inturrisi et al. 2022). Inturrisi et al. (2022) also mapped candidate resistance genes from the literature to the *B. juncea* reference genome and suggested *LMJR1* and *PhR2* were located on B03, and *LmJR2* on B08. Other studies have also found resistance sources in the three B-genome species which may or may not overlap with the reported *Rlm* genes. In hybrids between *B. carinata* and *B. napus*, resistance originating from *B. carinata* was independently associated with the middle to bottom of chromosome B3 and the top of chromosome B8 (Fredua-Agyeman et al. 2014).

The only mapped resistance locus in *B. nigra* so far is *Rlm10*, which was identified on chromosome B4 (Chèvre et al. 1996). Presence of this chromosome was found to confer resistance in *B. napus* × *B. nigra* hybrids (Roussel et al. 1999). However, Zhu et al. (1993) found that three chromosomes were associated with resistance in *B. napus-B. nigra* chromosome addition lines, each of which was then thought to correspond to a different genetic locus (Struss et al. 1996). So far, only one gene (*Rlm10*) has been identified in *B. nigra* on the basis of interactions with specific fungal isolates, but it is clear from previous studies that there are most likely multiple resistance genes which can be exploited from this species for rapeseed crop improvement.

Introgression breeding to transfer blackleg resistance (or any other trait) from *B. nigra* into *B. napus* (rapeseed) faces a number of challenges. Firstly, interspecific hybrids must be produced, potentially also overcoming pre- and post-fertilisation barriers which prevent fertilisation or embryo development (Chen et al. 2011, Katche et al. 2019). Generally, embryo rescue to produce viable hybrids is required in this cross combination (FitzJohn et al. 2007). Interspecific hybrids must then be backcrossed to *B. napus* (possibly following chromosome doubling to restore some fertility in the interspecific hybrid) and checked for expression of resistance. For the cross *B. napus* by *B. nigra*, we expect most *B. nigra* chromosomes to be inherited without recombination with the A or C genomes in the interspecific hybrids (Gaebelein et al. 2019, Navabi et al. 2013). Finally, we would hope that physical crossing over between chromosomes belonging to the rapeseed and black mustard genomes could occur or be induced to produce recombinant chromosomes (A/B or B/C), which would allow the material to be more fully exploited for breeding without linkage drag from associated *B. nigra* genes on the same chromosome (Mason and Chèvre 2016). However, in order to screen for recombinant chromosomes carrying the locus of interest, we also ideally need to know which chromosome/s are likely carrying the resistance from the *B. nigra* parent, and if this resistance corresponds to a known resistance locus or *AvrLm* gene.

Previously, we produced *B. napus* × *B. nigra* interspecific hybrids (2n = ABC), induced these hybrids to form allohexaploids (AABBCC), and established that these hybrids show blackleg resistance from the *B. nigra* parent (Gaebelein et al. 2019). Despite low fertility in these hybrids, backcrossing of the allohexaploids to *B. napus* successfully produced BC_1_F_1_ lines with several different *B. napus* cultivars. In the current study, we checked for presence of blackleg resistance genes in these *B. napus* accessions and carried out further backcrossing with lines where the resistance phenotype should originate solely from the *B. nigra* parent. We then investigated co-segregation of this blackleg resistance in BC_2_F_1_ plants with inheritance of each of the B-genome chromosomes (B1 - B8) using chromosome-specific markers that we designed and tested to indicate presence or absence of each of these chromosomes in the progeny. Based on these results and the presence of a hypersensitive response indicative of a major gene resistance, we identified that chromosome B2 of *B. nigra* is most likely carrying a previously uncharacterised major resistance gene locus for blackleg resistance.

## Materials and methods

### Parent species genotypes and plant growth conditions

The genotypes used in this study were *B. napus* accessions “Westar_010DH” (N2), “Darmor” (N14), and “Yudal” (N16) provided by the Australian Grains Genebank; *“*Boomer” (N5) and “Argyle” (N10) provided by Canola Breeders Western Australia; “Ningyou7” (N8) provided by Huazhong Agricultural University, China; “Express 617” (N9) a cultivar produced by NPZ GmbH, Hohenlieth, Germany and provided by Dr. Christian Obermeier, Justus-Liebig University Giessen, Germany and; “MSL 007 C” (N15) provided by NPZ Innovations GmbH, Hohenlieth, Germany. The *B. nigra* genotypes NGB 21858.1 and NGB 23253.2 (sourced from the Nordgen germplasm bank), are referred respectively as IX7 and IX13 in this paper, and the accession “Junius” was provided by Prof. Anne-Marie Chèvre, INRAE Le Rheu, France.

**Fig. 1:**
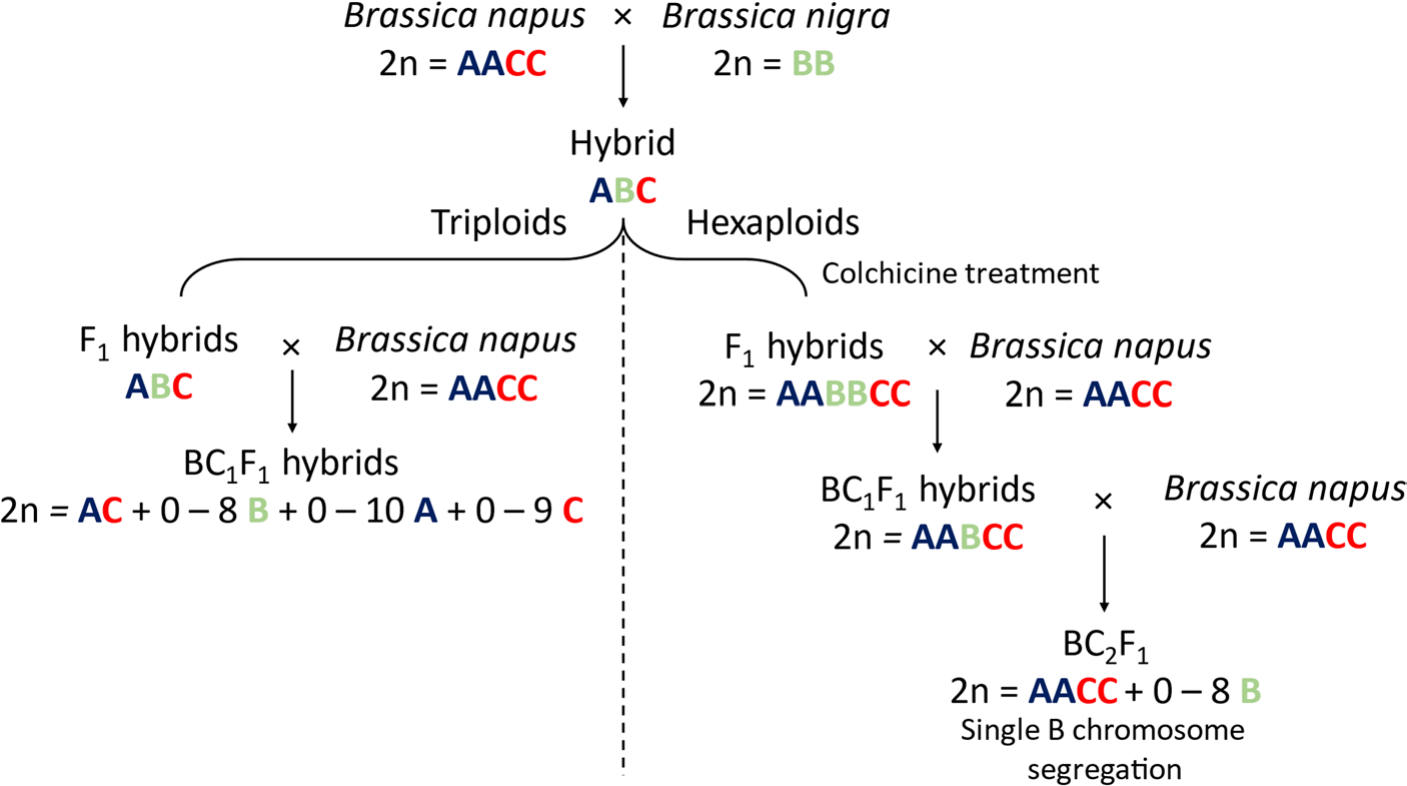
Crossing scheme for production of triploid and hexaploid lines by crossing *Brassica napus* × *Brassica nigra*, and their selfing (S) and backcrossing (BC). Material up to the BC_1_F_1_ generation is described in Gaebelein et al. (2019)

For this study, several *B. napus* × *B. nigra* cross combinations were used to produce the experimental material (Fig. 1). First, triploid hybrids (2n = ABC) and BC_1_F_1_ (*B. napus* × *B. nigra* × *B. napus*, 2n = AABCC) plants produced by Gaebelein et al. (2019) were grown under the same published glasshouse-controlled temperature and light conditions at Justus Liebig University Giessen, Germany, between February and December 2019 to produce a BC_2_F_1_ generation via backcrossing to *B. napus*. Then, the BC_2_F_1_ plants were grown under glasshouse conditions at the University of Bonn, Germany, during December 2020 to August 2021, with supplemental lighting to maintain a 16 h photoperiod. Both BC_1_F_1_ and BC_2_F_1_ plant generations were transplanted to the respective glasshouse conditions after testing them for resistance against blackleg, around 28 days after sowing (growing conditions as described in the cotyledon test section).

### Cross- and self-pollination of triploid plants

To determine the fertility of the triploid plants, two cuttings from each triploid genotype were used: “N15 × Junius”, genotypes Tpl-1 and Tpl-2; “N15 × IX7”, genotypes Tpl-3 and Tpl-4; and “N15 × IX13”, genotypes Tpl-5 and Tpl-6 (codes described in Supplementary Table 1). To encourage the production of seeds, triploid inflorescences were bagged together with inflorescences of the *B. napus* N2 and N14. Pollination bags were frequently shaken during the flowering period. Once the flowering ended, but the siliques were still green, the inflorescences from the triploids and *B. napus* parents were separated, keeping only the inflorescence from the triploid bagged. Seeds were then collected from the triploid plants.

### Production of BC_2_F_1_ and BC_1_S_1_ from BC_1_F_1_ (pentaploid, 2n = AABCC)

In this study, 14 maternal BC_1_F_1_ plants were selected for crossing based on higher seed fertility (Supplementary Table 2): two from the cross (N15 × IX13) × N5 (lines BC_1_F_1_−1a and BC_1_F_1_−1b), three from the cross (N15 × IX13) × N8 (lines BC_1_F_1_−2a, BC_1_F_1_−2b, and BC_1_F_1_−2c), five from the cross (N5 × IX7) × N8 (lines BC_1_F_1_−3a, BC_1_F_1_−3b, BC_1_F_1_−3c, BC_1_F_1_−3d, and BC_1_F_1_−3e), and four from the cross (N5 × IX7) × N14 (lines BC_1_F_1_−4a, BC_1_F_1_−4b, BC_1_F_1_−4c, and BC_1_F_1_−4d). From each of the 14 plants, three cuttings were taken to facilitate the crossing. An average of 100 manual cross pollinations were carried out between each maternal plant genotype and N2, N9, and N14 to produce BC_2_F_1_ seeds. Self-pollination of the BC_1_F_1_ plants was also carried out to produce BC_1_S_1_ seeds via (1) manual bud pollination, using an average of 100 buds for those plants that by visual assessment showed more pollen in the anthers, and (2) by bagging three flowering racemes per clone. All manually cross-pollinated and self-pollinated racemes were covered by microperforated plastic bags to avoid natural cross pollination.

### *Leptosphaeria maculans* inoculum preparation

Two isolates were used to test cotyledon-stage blackleg disease resistance in the interspecific hybrids: IBCN02 (T12aD34) containing *AvrLm4-7*, *AvrLm5-9*, *AvrLm6* and *AvrLm8* (presence of *AvrLm10a/b* unknown) belonging to the International Blackleg of Crucifers Network (IBCN) collection, provided by Dr. Birger Koopmann, Universität Göttingen, and described by Balesdent et al. (2005) and the isolate JN2 (v.23.1.2) containing *AvrLm4-7*, *AvrLm5-9*, *AvrLm6*, *AvrLm8*, *AvrLm10A-B*,* AvrLmS-Lep2*, and *AvrLm11* (provided by Université Paris-Saclay, INRAE, UR BIOGER, France).

The pycnidiospore inoculum was produced as described by Ansan-Melayah et al. (1995) and the spore suspension was adjusted to 107 spores ml^–1^ using a Malassez cell, aliquoted into 2 ml Eppendorf tubes and immediately stored at − 20 °C.

### Resistance testing in parent *B. napus* and *B. nigra* accessions

To carry out BC_1_F_2_ crosses for mapping population, it was necessary to phenotype parental lines N2, N5, N8, N9, N10, N14, N16, IX7, IX13, and Junius to determine the presence of resistance genes. This was done at INRAE, UR1290 BIOGER, Thiverval-Grignon, France, under the supervision of the group ‘Effectors and Pathogenesis in *Leptosphaeria maculans’* (EPLM) during January to February 2020. These genotypes were tested using the protocol of Balesdent et al. (2001) in four independent experiments using different sets of inoculums in each experiment (Supplementary Table 3). Here, it was found that N2 and N8 did not show resistances to these inoculums, whereas other genotypes presented phenotypes consistent with the presence of one or more known resistance genes or other unknown resistances. N5 presented with an unknown resistance gene (putatively *Rlm6*, which is thought to originate from *B. juncea*) and N14 with *Rlm4*. *Brassica nigra* IX7 and IX13 presented high resistance thanks to possible new unknown resistance genes, together with the possible presence of *Rlm6*. The genotype N15 was tested by Gaebelein et al. (2019) where it did not show resistance genes at the cotyledon stage but did show resistance in a stem inoculation test. Therefore, we selected BC_1_F_1_ cross combination (N15 × IX13) × N8 × N2, with no identified resistances from the *B. napus* parents involved, to identify the chromosomal origin of the resistance in IX13.

### BC_1_F_1_ cotyledon tests

Four BC_1_F_1_ genotype combinations were initially selected on the basis of seed fertility (Gaebelein et al. 2019): (N15 × IX13) × N5, (N15 × IX13) × N8, (N5 × IX7) × N8 and (N5 × IX7) × N14. Six to seven seedlings for each of these four BC_1_F_1_ genotypes (24 seeds in total) were inoculated at cotyledon stage with IBCN02 spores and grown under controlled growth room conditions as described by Balesdent et al. (2001) (Supplementary Fig. 1). These experiments were done at Justus-Liebig-University Giessen, in February 2019. IBCN02 (T12aD34) was an inoculum selected due to its containing *AvrLm4-7*, *AvrLm5-9*, *AvrLm6* and *AvrLm8* (presence of *AvrLm10a/b* unknown) and known virulence to *RLm1*,* RLm2-LepR3*,* RLm3*, and *RLm4-7–9.* This genotype was selected due its ability to infect the *B. napus* parent, coupled with the resistance of *B. nigra* IX13 to this isolate which was previously tested in Gaebelein et al. (2019).

### BC_2_F_1_ cotyledon tests

Only BC_2_F_1_ plants from the cross combination (N15 × IX13) × N8 × N2 (*B. napus* parents MSL, Westar_10DH and Ningyou7) were tested for resistance with isolate JN2, as all other BC_2_F_1_ cross combinations had some level of resistance in their *B. napus* parents after phenotyping for resistance at INRAE, France (Supplementary Table 3). JN2 (v23.1.2) of *Leptosphaeria maculans* was also selected due to its aggressiveness, which ensures a HR within the 13 dpi testing period. In addition, its genotype, symptom development and interaction mechanism behaviour are documented in the literature, with JN2 thought to contain containing *AvrLm4-7*, *AvrLm5-9*, *AvrLm6*, *AvrLm8*, *AvrLm10A-B*,* AvrLmS-Lep2*, and *AvrLm11* (Van De Wouw et al. 2023). This isolate was validated across all parental lines prior to this current experiment (Supplementary Table 3). The inoculations with JN2 were carried out at the University of Bonn under the same controlled growth cabinet conditions. The IMASCORE rating scale (Balesdent et al. 2001) was used to score symptoms 13 days post-inoculation (dpi), where scores of 1 to 3 were considered as a resistance response of the plants, and plants with of scores 4 to 6 were considered susceptible.

### DNA extraction and molecular marker genotyping

Leaf samples were collected in 2 ml microcentrifuge tubes and stored at −20 °C until use. DNA from the BC_1_F_1_ population was extracted from young leaf tissue using the CTAB method (Doyle and Doyle 1990). The DNA quality was confirmed with agarose gel electrophoresis, and DNA was quantified with a Qubit 2.0 fluorometer (Life Technologies, Darmstadt, Germany) before SNP array genotyping. The BC_2_F_1_ DNA extraction was done using the CTAB method, DNA quality was checked with agarose gel electrophoresis, and DNA concentration was estimated using a Nanodrop spectrophotometer 200c (Thermo Fisher Scientific Inc., Waltham, USA).

A subset of 24 BC_1_F_1_ individuals (2n = AABCC) was genotyped using the Illumina Infinium 90 K *Brassica* SNP array to verify the presence of A- and C-genome chromosome pairs, as well as the presence of a single copy of each B-genome chromosome, and to check if any B-genome chromosomes were only partially present, indicative of non-homologous recombination. The *Brassica* 90 K Illumina Infinium array is for the A, B and C genomes; it was a later-developed array that added 30 K SNPs to the existing A and C genome array, which is the *Brassica* 60 K Illumina Infinium array (Clarke et al. 2016). More details about these arrays are available in Mason et al. (2017). In the BC_2_F_1_ (segregating for single B-genome chromosomes following the cross AABCC × AACC) inheritance of individual B-genome chromosomes was tested with one locus-specific PCR marker per chromosome from either previously published marker sets (Lagercrantz and Lydiate 1995, Axelsson et al. 2000, Mason et al. 2011) or newly designed from the published *B. nigra* Ni100 reference genome (Perumal et al. 2020) (Supplementary Table 4). In order to check the specificity of previously published markers, primer sequences were aligned using BLAST tool (http://brassicadb.cn/#/BLAST/) with default parameters to the *B. nigra* Ni100 v2 and *B. napus* Darmor-*bzh* v10 (Rousseau-Gueutin et al. 2020) reference genome sequences available at http://brassicadb.cn. The criteria for selection were as follows: (1) at least one of the forward or reverse primer sequences had to be specific to a single locus on one B chromosome, and (2) either no predicted products in the *B. napus* genome or only PCR products of a different fragment size relative to the B-genome PCR products. Primers were designed to either end of the B-genome chromosomes in the *B. nigra* Ni100 v2 sequence using primer3plus (www.bioinformatics.nl/cgi-bin/primer3plus/primer3plus.cgi), followed by a quality check with PCR Primer Stats (https://www.bioinformatics.org/sms2/pcr_primer_stats.html).

### Statistical analysis

Seed set data was analysed using Lilliefors (Kolmogorov-Smirnov) normality test, and as it did not fulfill the normality criterium, the non-parametric test Kruskal-Wallis test was performed. The p value was less than 0.05, therefore the analysis was done using Dunn’s test for nonparametric pairwise multiple comparisons in independent groups. All analyses and figures were done using the statistical program R version 4.4.0.

Inheritance of chromosome markers and association of markers with resistance were tested using Pearson’s Χ^2^ test (α = 5%), against expected Mendelian segregation ratios for major gene resistance (e.g. 1 : 1 resistant: susceptible for a single dominant resistance locus segregating in a BC_2_F_1_ population, as well as 1 : 1 for presence/absence of specific B genome chromosomes). The software Microsoft Excel from Microsoft 365 was used for statistical analyses and figures.

## Results

### Backcrosses of the ABC triploid to *B. napus*

Triploid genotypes from the six cross combinations (Gaebelein et al. 2019) were bagged together with two different *B. napus* cultivars, N14 (Darmor) and N2 (Westar). From these crosses, the combinations Tpl-1 × N14, Tpl-1 × N2, Tpl-3 × N2, Tpl-5 × N14, and Tpl-6 × N14 produced zero seeds. The crosses Tpl-3 × N14 and Tpl-4 × N14 produced one seed each, Tpl-4 × N2 six seeds, Tpl-2 × N14 15 seeds, Tpl-6 × N2 22 seeds, Tpl-5 × N2 70 seeds, and Tpl-2 × N2 145 seeds.

### Production of the allohexaploid-derived BC_1_S_1_ and BC_2_F_1_ progeny

BC_1_F_1_ generation plants (pentaploid, 2n = AABCC) were previously produced by crossing between allohexaploid lines and *B. napus* (Gaebelein et al. 2019). Subsequently, we produced a pentaploid BC_1_S_1_ generation by manual crossing and bagging of branches to encourage self-pollination. An average of 100 buds per genotype were manually self-pollinated within the same plant to overcome putative self-incompatibility issues. Plant BC_1_F_1_−4d produced four seeds from a total of 101 bud pollinations, BC_1_F_1_−3d one seed from 100 bud pollinations, and all other manually self-pollinated plants produced zero seeds. From bagged branches, BC_1_F_1_−4d produced 16 seeds, BC_1_F_1_−3e produced 11 seeds, BC_1_F_1_−3a and BC_1_F_1_−2b produced one seed each, and all other genotypes produced zero seeds.

**Fig. 2:**
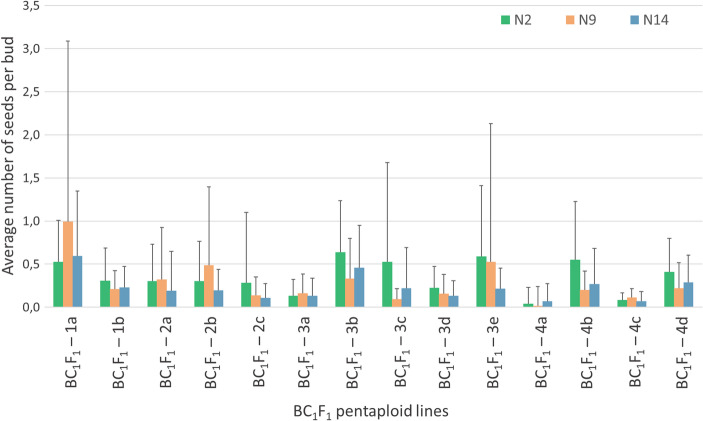
Seeds produced per bud pollination between different genotypes of interspecific *B. napus* × *B. nigra* pentaploid hybrids in backcrosses with *B. napus* (cultivars N2, N9 and N14)

BC_2_F_1_ progenies were produced by manual crossing between the BC_1_F_1_ pentaploid (2n=AABCC) as the mother, and three different *B. napus* cultivars as the fathers (Fig. 2). Overall, 0.4 seeds were produced per bud pollination on average. Developed pods with aborted seeds were frequently observed. No statistical differences in crossing success were observed between different fathers (N2 vs. N9 vs. N14).

### Resistance screening in the parent genotypes and non-segregating hybrid generations

All BC₁F₁ plants tested exhibited resistance to the IBCN02 isolate (*AvrLm4-7*, *AvrLm5-9*, *AvrLm6* and *AvrLm8* - presence of AvrLm10a/b unknown), regardless of genotype combination (Supplementary Fig. 1). Based on these results, together with the phenotypic evaluation of the parental lines, the cross (N15 × IX13) × N8 × N2 was selected because it lacked the N5 genotype, which conferred a novel type of resistance. This cross was chosen since all individuals derived from *B. napus* were susceptible to the fungus.

### Inheritance of blackleg resistance based on phenotyping results

**Fig. 3:**
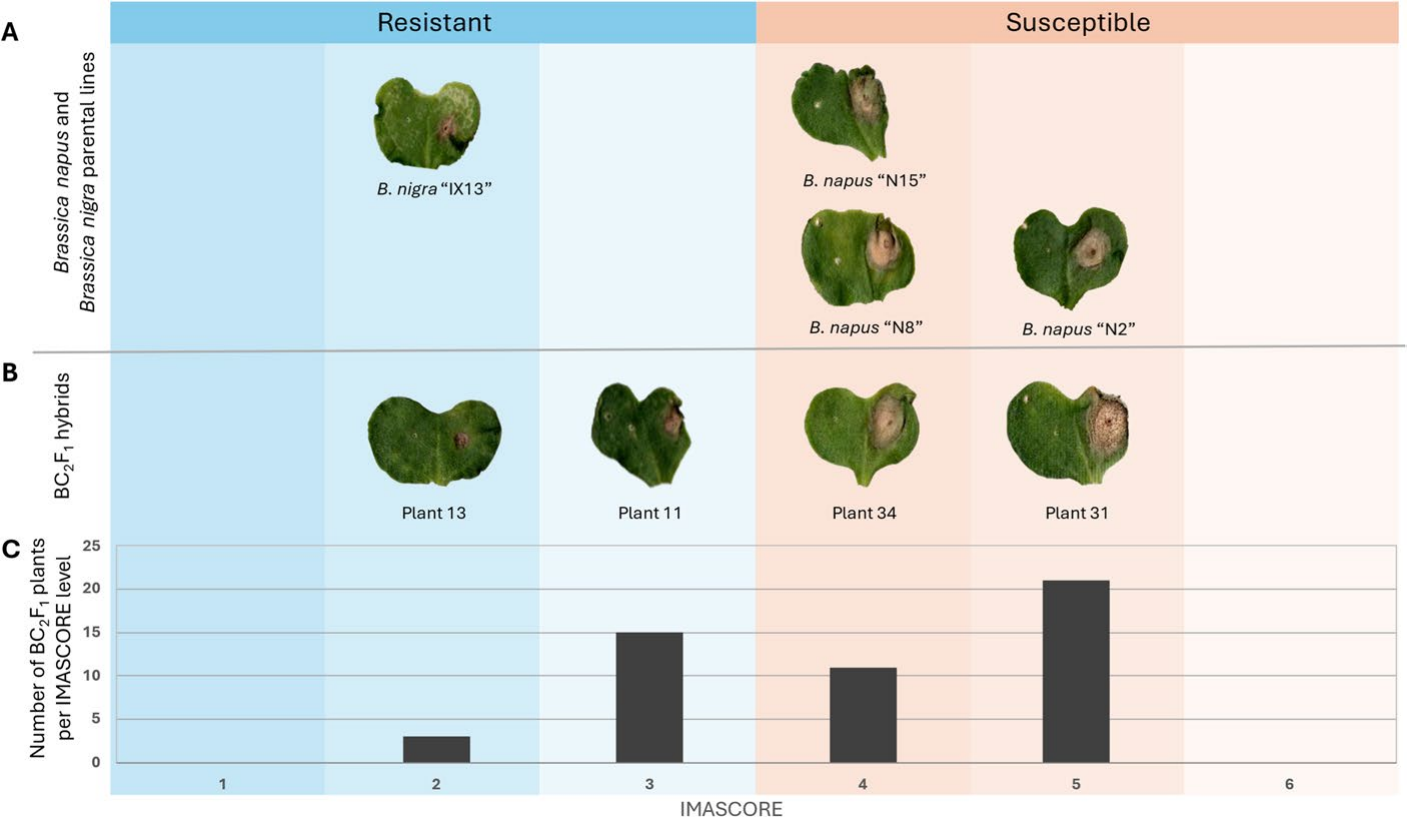
Phenotypic evaluation of *Leptosphaeria maculans* resistance in *Brassica nigra*, *Brassica napus*, and BC_2_F_1_ hybrids. (A) Parental lines; (B) Representative BC_2_F_1_ hybrids; (C) Distribution of IMASCORES in the BC_2_F_1_ population

A BC_2_F_1_ population predicted to be segregating for resistance (Fig. 3) was selected for phenotyping and genotyping. This population was derived from the cross combination (N15 × IX13) × N8 × N2, where all *B. napus* parents involved (N15, N8 and N2) have no known resistance to blackleg, such that all resistance in this cross combination should originate from the IX13 *B. nigra* parent. BC_2_F_1_ plants are expected to be segregating for presence or absence of individual B-genome chromosomes, as these are derived from the cross of the BC_1_F_1_ parent (2n = AABCC) with *B. napus* (AACC).

Out of a total of 50 BC_2_F_1_ plants tested, 18 plants showed resistance and 32 showed susceptibility, based on the observed phenotypes (Fig. 3). The presence of resistance was determined by the size of the lesion, but especially by the presence of a black margin around the lesion, as this indicates a hypersensitive reaction of the plant against JN2. In the case of susceptible plants, this response is not visible and did not limit the spread of the symptom, such that over time, it covers the whole half of the cotyledon where the inoculation was carried out, and even in some cases produced pycnidiospores.

### Inheritance of B-genome chromosomes

A set of 24 BC_1_F_1_ (AABCC) plants were genotyped using the *Brassica* Illumina Infinium 90 K SNP array: of these, one plant was missing one B-genome chromosome (B6), and for all other plants all other B-genome chromosomes were present and intact, with no indication of B/A-C recombination events. Chromosome-specific PCR markers were used to assess presence of each B-genome chromosome in each plant of the BC_2_F_1_ B-genome segregating generation (Supplementary Table 5). As the BC_2_F_1_ plants were derived from the cross AABCC × AACC, with only one copy of each B-genome chromosome present in the maternal parent plant in the backcross, the theoretical probability of inheritance for each chromosome was 50% (Table 1 and Supplementary Fig. 2). However, only two chromosomes – B4 and B5 – were inherited approximately 50% of the time in the BC_2_F_1_ progeny, as expected (present in 19/50 and 22/50 plants respectively, Pearson’s chi-squared test for count data, *p* > 0.05). Most commonly, B-genome chromosomes were present in the progeny less often than expected by chance: this was the case for chromosomes B2 (16/50 plants, *p* = 0.01), B3 (13/50 plants, *p* < 0.001), B6 (8/50 plants, *p* < 0.0001), B7 (6/50 plants, *p* < 0.0001) and B8 (10/50 plants, *p* < 0.0001). In contrast, chromosome B1 was present more often than expected by chance (42/50 plants, *p* < 0.0001).

**Table 1:**
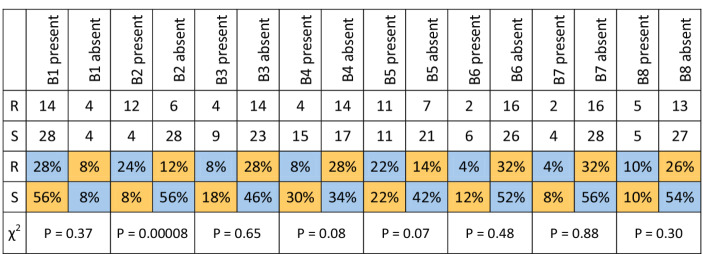
Plants (number and percentage) from the BC_2_F_1_ population segregating for presence/absence of each B-genome chromosome and for resistance/susceptibility to blackleg isolate JN2

Over-representation of resistant individuals which have a copy of that chromosome and/or individuals which are susceptible and missing a copy of that chromosome (blue shading) suggests that presence of the chromosome is associated with resistance. P-values were calculated using Pearson’s χ2 test

## Discussion

In this study, we investigated interspecific hybrid progeny from the cross *B. napus* × *B. nigra* followed by two generations of backcrossing for presence of specific B-genome chromosomes and blackleg disease resistance at the cotyledon stage, and identified segregation of resistance with presence of chromosome B2. The correspondence observed was significant but not exact, suggesting either environmental influence or other genetic factors are also present, but our results suggest for the first time that a major gene locus for blackleg resistance is present on chromosome B2 of *B. nigra*. The lines used in this study can further be used as advanced material for introgression of this blackleg resistance locus into *B. napus* for rapeseed crop improvement.

Production of self-pollinated seeds via bagging and via hand pollination was not very successful in these interspecific hybrids. In most cases, self-pollination both by bagging the entire inflorescence and by hand-pollination of buds did not produce seeds. We expected that encouraging self-pollination by bagging inflorescences might not be successful due to possible expression of self-incompatibility alleles from the *B. nigra* parent. *Brassica nigra* has sporophytic self-incompatibility (Becker et al. 1999), which means that it recognises the S alleles of the mother plant that are present in the pollen sporodermis, such that pollen from the same plant is not able to germinate and fertilise ovules on this plant. However, hand-pollination of immature stigmas (before bud maturation) is known to allow self-pollination to occur in *Brassica*, as the pollen recognition factors in the stigma are not yet active (Novikova et al. 2022, Goring et al. 2023). Despite this and regardless of the method used, few self-pollinated seeds were obtained. This may suggest post-fertilisation barriers (e.g. to embryo development), or other incompatibility or fertility issues in the hybrids, which is unfortunate but not common in interspecific *Brassica* crosses (Chen et al. 2011, Katche et al. 2019, Gaebelein and Mason 2018).

Cross-pollination between the interspecific pentaploid hybrids as the mother and *B. napus* as the father was difficult but more successful in producing seeds than self-pollination. The average success rate was low, with less than one seed per bud pollination: in fertile canola *B. napus* plants an average of 15–40 seeds per silique is expected (Canola 2024). Possibly, this low seed production rate and the frequent observation of aborted seeds indicate genetic factors present in the *B. nigra* genome, which hinder or stop the development of the embryos post-pollination, although the exact mechanism/s responsible are unknown. As well, environmental factors such as heat waves in summer over part of the crossing period (approximately one year) may have had negative impacts on crossing success, as heat is known to affect pollen viability and germination, induce silique abortion and decrease seed production (Lohani et al. 2022).

Clear phenotypic segregation between resistant and susceptible plants is critical for mapping resistances. To determine the heritability of *B. nigra*-derived blackleg resistance in our interspecific hybrids, we used a BC_2_F_1_ segregating population from the combination (N15 × IX13) × N8 × N2 where the *B. napus* parents have no known resistance. In this case, distinct resistance and susceptibility symptoms were clearly observable following inoculation with isolate JN2 (Fig. 3), specifically as the presence or absence of a black margin around the lesion. This black margin corresponds to a hypersensitive reaction (HR) that produces local cell death due to Effector Triggered Immunity (ETI), thus halting the progression of the infection (Vasquez-Teuber et al. 2024). Based on this phenotypic observation, we can predict that blackleg inheritance in our population corresponds to a gene-for-gene model interaction, where a resistance gene in the host reacts with the corresponding avirulence gene in the pathogen (Flor 1955) as it has been commonly found in this pathosystem with numerous *Brassica* species (Rouxel et al. 2024). Thus, our phenotyping results support major gene resistance in the B genome, as also predicted by previous studies (Chèvre et al. 1996). However, the presence of other chromosomes or possible non-homologous recombination events might influence the resistance response, since some individuals with the B2 marker do not show resistance, whereas some without the B2 marker do show resistance. As well, inaccuracies in assessment of the hypersensitive response are not impossible based on single phenotypic observations. To confirm these findings, it would be beneficial to study larger populations of interspecific hybrids (although these are difficult to obtain) or to carry out genetic mapping within *B. nigra.*

Chromosome B2 has not previously been associated with blackleg resistance, although to date relatively few studies have investigated *B. nigra* or B-genome species *B. juncea* and *B. carinata* for blackleg resistance. Isolate JN2 is known to contain each of *AvrLm5-9*, *AvrLm6* and *AvrLm10A-B* (Van De Wouw et al. 2023) and hence we cannot rule out that our locus represents *Rlm5*, *Rlm6* or *Rlm10*. In *B. juncea*, *Rlm6* was putatively mapped to several B-genome chromosomes, but not to B2 (Yang et al. 2021), while *Rlm5* has been described in *B. juncea* and *B. rapa* but not localised to the chromosome level (Vasquez-Teuber et al. 2024). Previous mapping work has suggested that *Rlm10* (at least one copy) is located on chromosome B4 (Chèvre et al. 1996). However, we cannot rule out the possibility of mismapping in either our study or this study, or even that multiple copies of the same R genes may be present on different chromosomes in *B. nigra*. In future, this should be further validated in populations of *B. nigra*, where fine-mapping of resistance loci can be done, as opposed to in interspecific hybrids which inherit primarily whole B-genome chromosomes. On the other hand, it is highly likely that uncharacterised sources of blackleg resistance are present in each of *B. nigra*, *B. juncea* and *B. carinata*, and these sources are already attracting attention as a source of novel variation for rapeseed improvement via production of synthetic allohexaploids (Shah et al. 2026).

Regarding the heritability of the B genome in the BC_2_F_1_ generation, a 50% probability of heritability for each B chromosome was expected in the BC_2_F_1_ segregating population, as only one copy of each B genome chromosome existed in the BC_1_F_1_ maternal plant, which was then backcrossed with *B. napus* (AABCC × AACC). However, only chromosomes B4 and B5 segregated as expected: B1 was inherited more often than expected by chance and B2, B3, B6, B7 and B8 were inherited less often than expected by chance. The mechanism for this biased transmission of univalent chromosomes is unknown, although this may be related to selection for viable progeny in the cross combination, e.g. affecting the survival of hybrid seeds (Gaeta and Pires 2010). Similar results have also been observed in previous studies of *Brassica* interspecific hybrids, including elimination of the B genome following self-pollination of AABCC hybrids derived from the cross *B. rapa* × *B. carinata* (Meng et al. 1998), more frequent elimination of the B genome relative to A and C genomes in CC hybrids (Ge et al. 2009) and in crosses between *B. napus* × *B. juncea* (F_1_ = AABC) elimination of the B genome by self-pollination and backcrossing with *B. napus* (Schelfhout et al. 2006).

We did not observe any partial inheritance of B-genome chromosomes indicative of introgression based on our Illumina Infinium 90 K SNP array analysis of inheritance of the B-genome chromosomes in the pentaploid AABCC hybrid. This result is disappointing, as non-homologous recombination between the B-genome and A/C-genome chromosomes is required to physically introgress B-genome loci of interest into an elite rapeseed (2n = AACC) genome background without linkage drag (associated traits with negative agronomic effects on the same chromosome as the desirable resistance locus) but is not surprising. B-A/C recombination has previously been quantified in *B. napus* by *B. nigra* ABC triploids and AABBCC allohexaploids as approximately one event per three pollen mother cells and one event per six pollen mother cells respectively (Gaebelein et al. 2019). Following segregation of these chromosomes into daughter cells after meiosis, only approximately 1/12 or so of our AABCC lines would be expected to contain recombinant B-A/C chromatids, such that 24 plants may not have been enough to see any recombination events. Putatively, larger populations of interspecific hybrids could be recovered following further backcrossing and selection for chromosome B2 to improve the chances of recovering the desired recombinant, or alternatively methods such as radiation treatment (Agrawal et al. 2021) or in future even nuclease-induced recombination (Beying et al. 2020) could be used to facilitate the desired chromosome introgression for breeding purposes.

## Supplementary Information

Below is the link to the electronic supplementary material.

Supplementary Table 5 (XLSX 11.9 KB)

Supplementary Table 2 (XLSX 10.6 KB)

Supplementary Figure 2 (PPTX 51.2 KB)

Supplementary Table 3 (XLSX 14.2 KB)

Supplementary Figure 1 (PPTX 11.8 MB)

Supplementary Table 1 (XLSX 10.8 KB)

Supplementary Table 4 (XLSX 10.3 KB)

## Data Availability

All information is available in the manuscript and supporting information.
