# Supplementary material for: Segregating BC2F1 interspecific hybrids between Brassica napus and B. nigra reveal a major effect locus for blackleg resistance on chromosome B2
Source: Mol Breed. 2026 Jul 4;46(7):69. doi: 10.1007/s11032-026-01690-5 (PMC13332926; doi:10.1007/s11032-026-01690-5)
Supplement: Supplementary file 3 — Supplementary Figure 2 (PPTX 51.2 KB) [file 11032_2026_1690_MOESM3_ESM.pptx]

## Slide 1
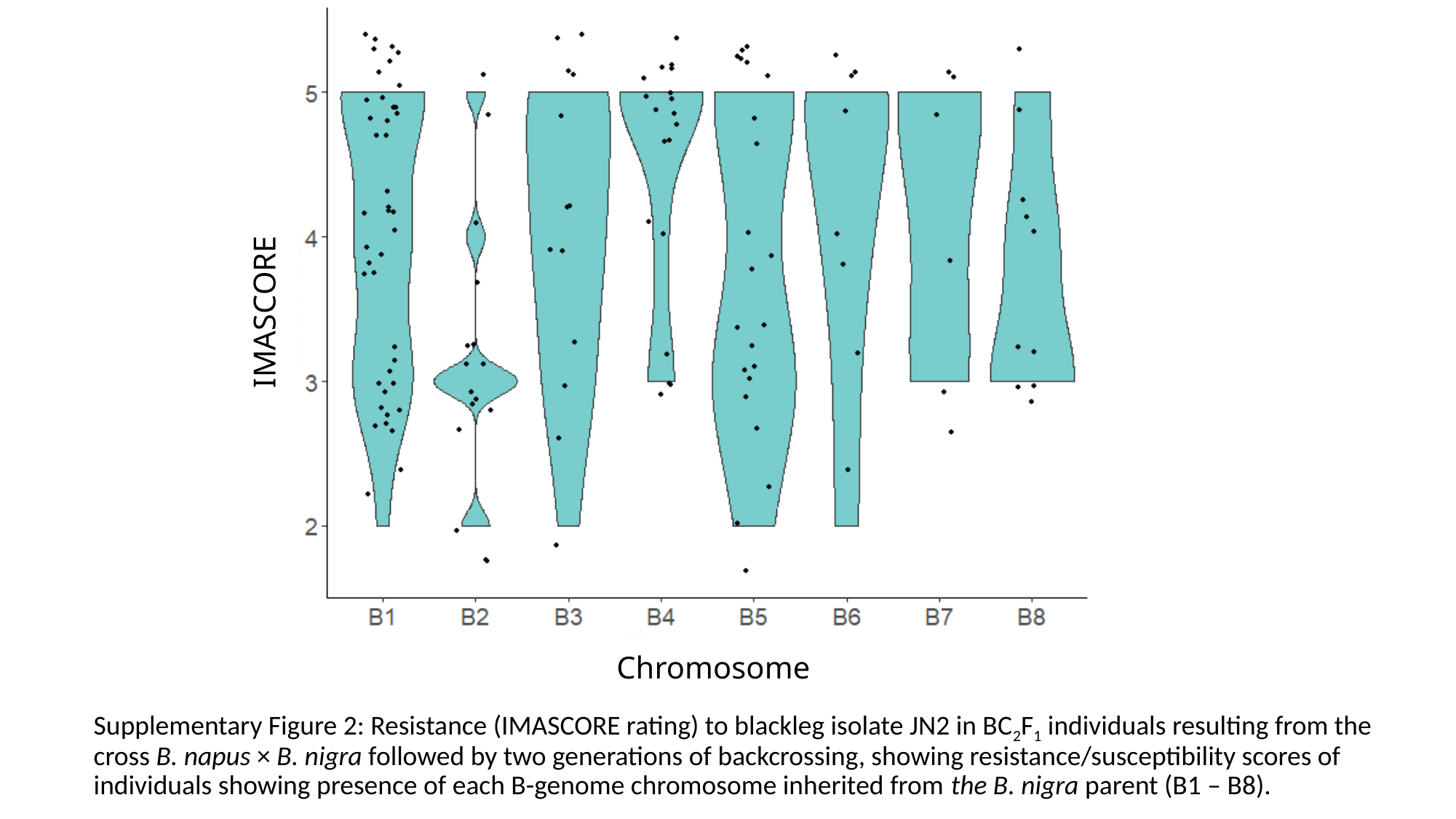

IMASCORE
Chromosome
# Supplementary Figure 2: Resistance (IMASCORE rating) to blackleg isolate JN2 in BC2F1 individuals resulting from the cross B. napus × B. nigra followed by two generations of backcrossing, showing resistance/susceptibility scores of individuals showing presence of each B-genome chromosome inherited from the B. nigra parent (B1 – B8).
