# Supplementary material for: Segregating BC2F1 interspecific hybrids between Brassica napus and B. nigra reveal a major effect locus for blackleg resistance on chromosome B2
Source: Mol Breed. 2026 Jul 4;46(7):69. doi: 10.1007/s11032-026-01690-5 (PMC13332926; doi:10.1007/s11032-026-01690-5)
Supplement: Supplementary file 5 — Supplementary Figure 1 (PPTX 11.8 MB) [file 11032_2026_1690_MOESM5_ESM.pptx]

## Slide 1
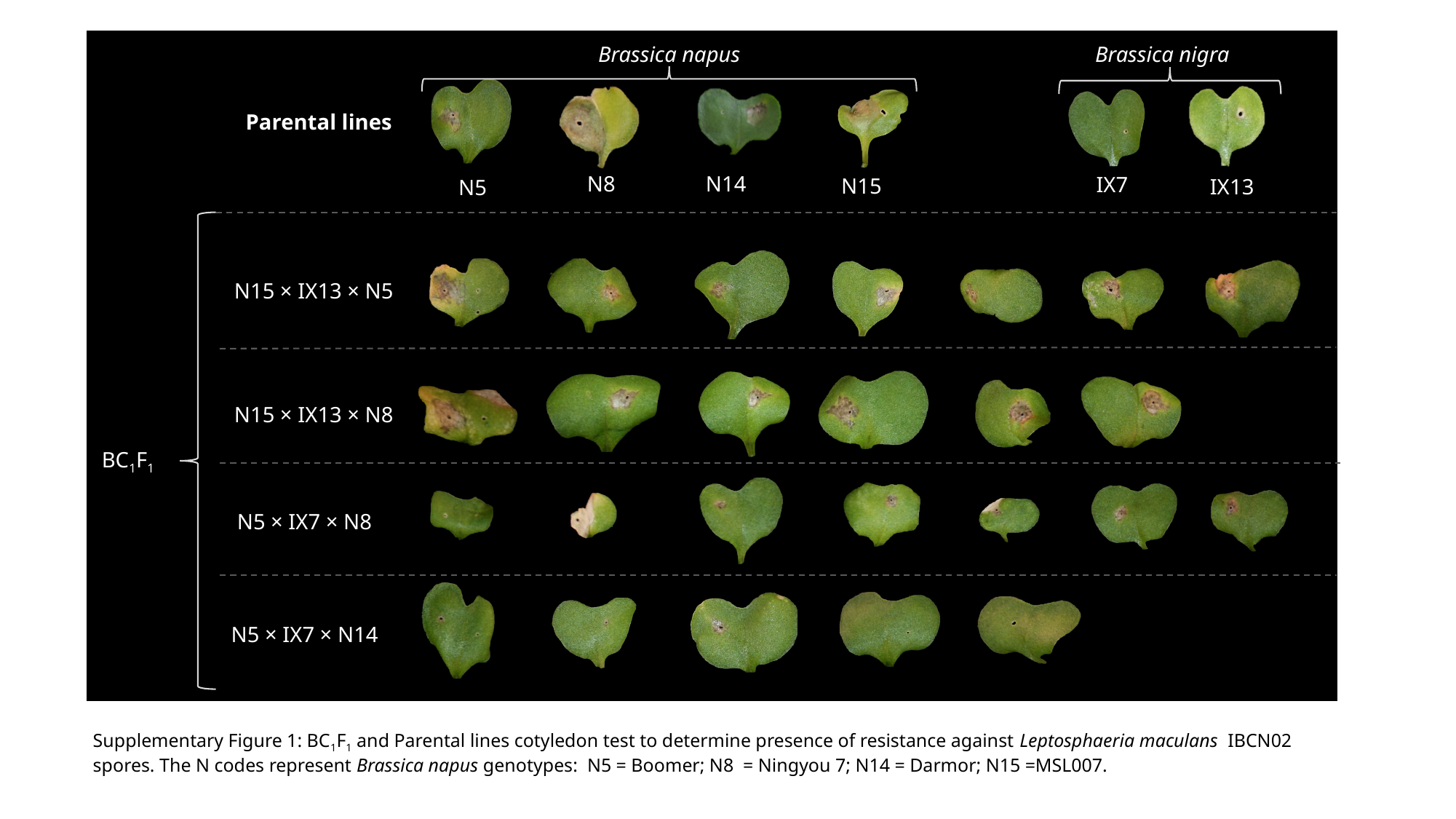

Brassica napus
Brassica nigra
IX13
IX7
Parental lines
N8
N14
N15
N5
N15 × IX13 × N5
N15 × IX13 × N8
BC1F1
N5 × IX7 × N8
N5 × IX7 × N14
Supplementary Figure 1: BC1F1 and Parental lines cotyledon test to determine presence of resistance against Leptosphaeria maculans IBCN02 spores. The N codes represent Brassica napus genotypes: N5 = Boomer; N8 = Ningyou 7; N14 = Darmor; N15 =MSL007.
